# Nitrogen fertilization coupled with iron foliar application improves the photosynthetic characteristics, photosynthetic nitrogen use efficiency, and the related enzymes of maize crops under different planting patterns

**DOI:** 10.3389/fpls.2022.988055

**Published:** 2022-09-02

**Authors:** Jamal Nasar, Gui-Yang Wang, Shakeel Ahmad, Ihsan Muhammad, Muhammad Zeeshan, Harun Gitari, Muhammad Adnan, Shah Fahad, Muhammad Hayder Bin Khalid, Xun-Bo Zhou, Nader R. Abdelsalam, Gamal A. Ahmed, Mohamed E. Hasan

**Affiliations:** ^1^Guangxi Key Laboratory of Agro-Environment and Agro-Products Safety, Guangxi Colleges and Universities Key Laboratory of Crop Cultivation and Tillage, Agricultural College of Guangxi University, Nanning, China; ^2^Department of Agricultural Sciences and Technology, Kenyatta University, Nairobi, Kenya; ^3^Department of Agriculture, University of Swabi, Swabi, Pakistan; ^4^Department of Agronomy, The University of Haripur, Haripur, Pakistan; ^5^National Research Center of Intercropping, The Islamia University of Bahawalpur, Bahawalpur, Pakistan; ^6^Agricultural Botany Department, Faculty of Agriculture (Saba Basha), Alexandria University, Alexandria, Egypt; ^7^Plant Pathology Department, Faculty of Agriculture, Moshtohor, Benha University, Benha, Egypt; ^8^Bioinformitics Department, Genetic Engineering and Biotechnology Research Institute, University of Sadat City, Sadat City, Egypt

**Keywords:** intercropping, photosynthetic rate, PNUE, nitrogen use efficiency, enzymatic activities, nitrogen, iron

## Abstract

Photosynthetic rate (Pn) and photosynthetic nitrogen use efficiency (PNUE) are the two important factors affecting the photosynthesis and nutrient utilization of plant leaves. However, the effect of N fertilization combined with foliar application of Fe on the Pn and PNUE of the maize crops under different planting patterns (i.e., monocropping and intercropping) is elusive. Therefore, this experiment was conducted to determine the effect of N fertilization combined with foliar application of Fe on the photosynthetic characteristics, PNUE, and the associated enzymes of the maize crops under different planting patterns. The results of this study showed that under intercropping, maize treated with N fertilizer combined with foliar application of Fe had not only significantly (*p* < 0.05) improved physio-agronomic indices but also higher chlorophyll content, better photosynthetic characteristics, and related leaf traits. In addition, the same crops under such treatments had increased photosynthetic enzyme activity (i.e., rubisco activity) and nitrogen metabolism enzymes activities, such as nitrate reductase (NR activity), nitrite reductase (NiR activity), and glutamate synthase (GOGAT activity). Consequently, intercropping enhanced the PNUE and soluble sugar content of the maize crops, thus increasing its yield compared with monocropping. Thus, these findings suggest that intercropping under optimal N fertilizer application combined with Fe foliation can improve the chlorophyll content and photosynthetic characteristics of maize crops by regulating the associated enzymatic activities. Consequently, this results in enhanced PNUE, which eventually leads to better growth and higher yield in the intercropping system. Thus, practicing intercropping under optimal nutrient management (i.e., N and Fe) could be crucial for better growth and yield, and efficient nitrogen use efficiency of maize crops.

## Introduction

Photosynthetic rate is an important index of the plants’ photosynthetic characteristics and photosynthetic nitrogen use efficiency (PNUE), which is referred to as the ratio of photosynthetic rate to leaf nitrogen (Zhong et al., 2019). It is an important indicator describing the plant’s leaf nutrient utilization capacity and physiological characteristics, which fully reflect the nitrogen allocation and the overall photosynthesis of the plant (Zhong et al., 2019; Nasar et al., 2021). The mutual proportion of the photosynthetic characteristics and photosynthetic nitrogen of the plant’s leaf has a direct effect on the PNUE of the plant (Hikosaka, 2004; Zhang Y. et al., 2017). The higher the photosynthetic rate, the higher the PNUE and the leaf nitrogen utilization rate of the plant (Ghannoum et al., 2005). Therefore, studying the photosynthesis and PNUE of the plant is an important mechanism to reveal its effect on the crops.

Nitrogen is a key constituent of the plant’s photosynthetic organ, which helps improve the chlorophyll content, enzyme content, and enzymatic activity of plant leaves, thereby promoting the plant’s photosynthesis system (Giersch and Robinson, 1987; Nasar et al., 2020b; Ochieng’ et al., 2021; Noor Shah et al., 2021). Existing studies have shown the relationship between nitrogen application rate on crops photosynthetic characteristics, nitrogen utilization rate, and crops yield (Vagusevičienė et al., 2013; Kong et al., 2016; Liu et al., 2019; Shah et al., 2021b). However, iron, which is a micronutrient is often required in small quantities, though it plays a major role in photosynthetic electron transport. It is one of the most important elements involved in chlorophyll formation, photosynthesis, and the photosynthetic enzyme (i.e., ribulose 1,5-bisphosphate carboxylase/oxygenase) in the plant (Wang et al., 2017; Yoon et al., 2019; Maitra et al., 2022). Iron also plays a pivotal role in nitrogen assimilation, uptake, and translocation by regulating the cofactor enzymes of nitrogen metabolism [i.e., nitrate reductase (NR), nitrite reductase (NiR), and glutamate synthase (GOGAT)] (Borlotti et al., 2012). As earlier reported, insufficient iron reduces the number of grana and stroma lamellae per chloroplast in plant leaves (Jiang et al., 2007; Yoon et al., 2019). In addition, its deficiency reduces the cumulative membrane components, including electron carders of the photosynthetic electron transport chain (Geider and La Roche, 1994; Karimi et al., 2019), light-harvesting pigments (Terry, 1983; Abadia et al., 1986), and nitrogen uptake (Borlotti et al., 2012).

Intercropping, which is the combined cultivation of at least two different plant species (i.e., cereal and legumes) is an ancient planting pattern plant (Maitra et al., 2020). It is mainly practiced to improve crops yield and better utilize the available resources (Gitari et al., 2018; Nyawade et al., 2020). Maize–soybean is a typical cereal–legume intercropping system, which has long been practiced in the subtropical and temperate regions of the world, because of some of the anticipated advantages, such as high yield production, better resources utilization (i.e., nutrient, light, water, and land), reduced risk of diseases and insect pests attack, and guaranteed environmental safety (Yong et al., 2012; Raza et al., 2020). It is well known that maize–soybean intercropping can improve the growth and yield of the crops and better utilize the available resources (i.e., water, light, nutrients, and land). However, due to the differences in plant height, these companion plants intercept sunlight in different directions, which inevitably changes their photosynthetic system (Jiao et al., 2013; Zhu et al., 2018). Moreover, the underlying interspecific competition for nutrients and the rhizosphere modifications that occur in maize–soybean intercropping systems (Ehrmann and Ritz, 2013; Zhang et al., 2013; Nyawade et al., 2021) can have a significant impact on post-intercropping nitrogen utilization (Nasar et al., 2021). This results in either under or overutilization of N, with a subsequent adverse effect on the plant photosynthesis capacity and leaf’s nutrient uptake and utilization use efficiency (Nasar et al., 2020b). However, such negative effects on the plant can be effectively reduced with appropriate nitrogen and iron application (Zhang et al., 2014; Pan et al., 2016).

Previously, the effect of nitrogen and iron fertilization on the chlorophyll and photosynthetic characteristics of the plants was only investigated in the monocropping system (Derviş et al., 2018; Liu et al., 2018; Karimi et al., 2019). Nevertheless, their combined effect on the aforementioned indices under intercropping system remains largely unknown. The detailed knowledge of the effect of nitrogen combined with foliar application of iron on crops photosynthesis and PNUE, particularly in intercropping, is missing and calls to be studied. Such a study is paramount mainly because the shading or interspecific competition that occurs in the intercropping system can be alleviated or even eliminated by either nitrogen or iron application. This is particularly so since shading and the underlying interspecific root competition for nutrients during intercropping are the two abiotic factors that adversely affect the physiology of the plant, which eventually leads to poor growth and subsequently low yield (Wu et al., 2017; Raza et al., 2019a).

Therefore, this study was designed to investigate the effect of nitrogen fertilization combined with iron foliar application on the chlorophyll, photosynthetic characteristics, photosynthetic nitrogen use efficiency, and associated enzymes of maize crops under different planting patterns (i.e., monocropping and intercropping). Such studies on the photosynthesis mechanism and photosynthetic nitrogen utilization efficiency of maize crops provide theoretical support and practical guidance for the rational regulation of nitrogen to take advantage of the growth and yield of maize during intercropping.

## Materials and methods

The experiment was conducted at the experimental area of Guangxi University, Nanning, China in the year 2021–2022. This area is characterized by a subtropical monsoon climate with an annual rainfall of 1,080 mm. The experimental soil was loam in texture having an organic matter of 23.7 g kg^–1^, total N of 0.118%, alkaline N of 109.9 mg kg^–1^, available P of 73.6 mg kg^–1^, available K of 79.0 mg kg^–1^, soil pH of 7.4, and available iron of 97.7 mg kg^–1^.

**FIGURE 1:**
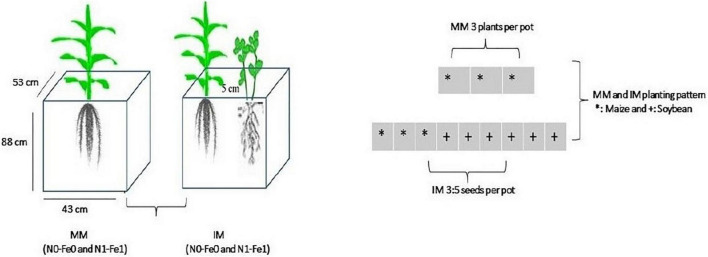
Schematic diagram of the experiment. MM, maize monocropping; IM, maize intercropping; N0-Fe0, no nitrogen and iron fertilization; N1-Fe1, nitrogen and iron fertilizers applications; *, maize crops and; +, soybean crops.

Maize crops (Ching Ching 700 variety) was planted as a monocrop (MM) and an intercrop (IM) with soybean crops (Gui Chun 15 variety) in large-sized pots (i.e., 88 cm height, 53 cm width, and 43 cm length) filled with 120 kg of soil (Figure 1). The pots, in four replicates, were randomly placed in a ventilated net house under natural light environment. Initially, five plants of maize crops and 10 seeds of soybean were planted in monocropping and in intercropping at a filed plant density of 60,000 maize plants ha^–1^ and soybean seed rate of 20 kg seeds ha^–1^, respectively. Later at the V3 growth stage, the maize and soybean plants were thinned to three and five (3:5) plants, respectively, in each pot to better adapt to the pot environment.

**FIGURE 2:**
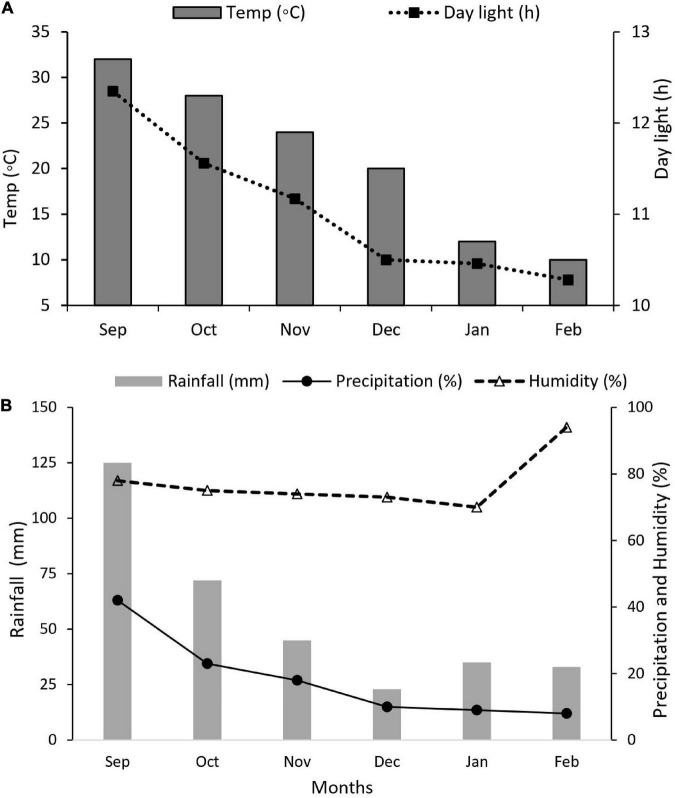
Weather forecast report during the experiment. **(A)** Temperature (°C) and daylight (h), **(B)** rainfall (mm), precipitation (%), and humidity (%).

Maize and soybean seeds were sown in mid-September 2021 and harvested in mid-February 2022. Nitrogen fertilizer was applied as soil dressing before sowing at the rate of 200 kg N ha^–1^. Iron (Fe) was sprayed on plant leaves at the rate of 0.15 mg Fe g^–1^ in three splits: at bell-mouthed stage, silking stage, and filling stage. The fertilizer arrangement and their combinations are shown in Table 1. In addition, basal doses of phosphorous and potassium fertilizers were applied uniformly to all experimental pots (i.e., P at 100 kg ha^–1^ and K at 100.0 kg ha^–1^). The sources of fertilizer used were urea (46% N), diammonium phosphate (P_2_O_5_ 46% P), potassium chloride (K_2_O 60% K), and iron as ferrous sulfate (FeSO_4_ 20.5% Fe). All the plants were watered normally, with weeds and insect pests being controlled with herbicides and pesticides, respectively, when needed. Different environmental factors such as temperature (°C), precipitation (%), rainfall (mm), humidity (%), and daylight (hrs) were carefully monitored and recorded (Figures 2A,B).

**TABLE 1:** The treatments combination of the experiment.

Treatment	Planting pattern	
	
Fertilizers application	MM	IM
N0-Fe0	No fertilization	No fertilization
N1-Fe0	Nitrogen fertilization without iron foliation	Nitrogen fertilization without iron foliation
N1-Fe1	Nitrogen fertilization with iron foliation	Nitrogen fertilization with iron foliation

### Data collection

#### Grain yield and biomass dry matter of maize crops

Crops harvesting was carried out at full maturity, whereby the plants were sun-dried, kernels removed and threshed to obtain the grains which were weighed using an electric scale to obtain the grain yield. The remaining plants’ stover was further oven-dried at 65°C for 72 to obtain biomass dry matter.

#### Chlorophyll and photosynthetic characteristics

At the V9 growth stage, the chlorophyll of maize leaves (fully expanded) was measured using the SPAD Chlorophyll Meter (SPAD-502, Minolta Camera, Tokyo, Japan) (Zhang et al., 2013). However, the different photosynthetic characteristics of the maize leaves (i.e., photosynthetic rate, stomatal conductance, transpiration rate, and intercellular CO_2_) were determined using a Li-6400XT portable photosynthesis system (LI-COR Inc., Lincoln, NE, United States) at leaf temperature of ∼27 °C. Nevertheless, the companion side leaves were selected to measure these parameters in the intercropping. These parameters were estimated in the morning between 9:00 am and 11:00 am, with the photosynthetic system being adjusted at a constant light of 80, 100, 150, 200, 400, 600, 800, and 1,000 μmol m^–2^ s^–1^ and a continuous CO_2_ level of 400 μmol mol^–1^ (Ahmad et al., 2013).

#### Maize leaf enzymes activities

After measuring the maize leaf photosynthesis at the V9 stage, the selected maize leaves were cut and frozen in liquid nitrogen to determine the plant’s leaf enzyme activities. Different enzymes such as the photosynthetic enzyme activity (rubisco) and the nitrogen metabolism enzymes activity, such as NR, NiR, and GOGAT, were determined using the plant enzyme kit from Sangon Biotech Co. Ltd. (Beijing, China) following the appropriate manual supplied with the kit.

#### Maize leaf traits measurement and calculation

The maize leaf area was measured with LI-3000C portable leaf area meter (LI-COR, NE, United States) at the V9 growth stage. The same leaf whose area was measured was plucked, sun-dried, and oven-dried at 80°C to constant weight to obtain the leaf dry mass (Eq. 1). The dried leaf samples were ashed at 105°C for 30 min and digested with a concentrated sulfuric acid–hydrogen peroxide mixture to obtain the nitrogen content in maize leaves using the Kjeldahl method (Jiao et al., 2013) and expressed per unit dry mass and unit area as indicated in Eqs 2, 3, respectively.


(1) $$\mathrm{Specific}\,\mathrm{leaf}\,\mathrm{mass}\,\left(\mathrm{LMA},g\,m^{2}\right)=\frac{\mathrm{Leaf}\,\mathrm{dry}\,\mathrm{mass}}{\mathrm{Leaf}\,\mathrm{area}}$$

(2) $$\mathrm{Leaf}N\mathrm{content}\mathrm{per}\mathrm{unit}\mathrm{dry}\mathrm{mass}\left({\text{N}}_{\mathrm{mass},}g.{\mathrm{kg}}^{1}\right)=\mathrm{leaf}\,N\,\mathrm{content}\times \mathrm{leaf}\,\mathrm{dry}\,\mathrm{mass}$$

(3) $$\mathrm{Leaf}N\mathrm{content}\mathrm{per}\mathrm{unit}\mathrm{area}\left({\text{N}}_{\mathrm{area},}\mathrm{mg}.{\mathrm{cm}}^{2}\right)={\text{N}}_{\mathrm{mass},}\times \text{LMA}$$

#### Calculation of photosynthetic nitrogen use efficiency to land equivalent ratio

Photosynthetic nitrogen use efficiency of the maize crops was calculated by simply multiplying the photosynthetic rate (Pn) by the nitrogen content per unit area (N_area_) (Eq. 4). The land equivalent ratio (LER) was computed as indicated in Eq. 5.


(4) $$\mathrm{PNUE}\,\left(\text{mol}\,m\,o\,l^{1}\,s^{1}\right)=\mathrm{Pn}\times {\text{N}}_{\text{area}}$$

(5) $$\mathrm{Land}\,\mathrm{Equivalent}\,\mathrm{Ratio}\,\left(\text{LER}\right)=\left(\frac{{\text{Y}}_{\text{im}}}{{\text{Y}}_{m\,m}}+\frac{{\text{Y}}_{\text{is}}}{{\text{Y}}_{\text{ms}}}\right)$$

where Y_im_ and Y_is_ represent the grain yield of maize and soybean crops in intercropping and Y_mm_ and Y_ms_ represent the grain yield of maize and soybean crops in monocropping. The LER is an indicator used to determine the competitiveness between intercrops for the available resources (Al-Dalain, 2009). If the value of LER is 1, it indicates that both monocrop and intercrop produce equal yield and utilize the available resources equally (Gitari et al., 2020). If the value of the LER is greater than 1, it suggests a greater complementary effect of intercropping maize than a competitive one, and produces a higher yield compared to monocropping. If the value of LER is less than 1, it indicates that interspecific competition is greater than interspecific facilitation, and there is no intercropping advantage. So, the higher the LER, the greater the benefit of increasing yield in intercropping over monocropping (Soratto et al., 2022).

#### Soluble sugars and starch quantification in maize leaf

The content of soluble sugars (i.e., sucrose, glucose, and fructose) in maize leaf content was determined by the anthrone colorimetric method and the starch iodine colorimetric method at the V9 growth stage. For soluble sugars, 0.2 g of the minced fresh leaf of the maize crops was boiled in 5 ml of distilled water. This process was repeated for about 30 min to collect 25 ml of extract. Then, 0.125 ml of extraction solution was thoroughly mixed with 1.87 ml of distilled water, 0.5 ml of anthrone ethyl acetate reagent, and 5 ml of concentrated sulfuric acid. The mixture was boiled in boiling water for 1 min after which it was cooled at room temperature and the content of soluble sugar was determined by a TU-1900 spectrophotometer at 630 nm (Du et al., 2020). For starch content, 0.5 g sample of fresh maize leaf was minced with 2 mL of distilled water and 3.2 ml of 60% of perchloric acid. The above portion of the solution was collected and centrifuged at 5,000 *g* for 5 min. About 0.5 ml of the supernatant (mixed with 3 mL of distilled water and 2 ml of iodine reagent and the starch content) was determined by spectrometry at 660 nm (Kuai et al., 2014).

### Data analysis

The data were computed and formulated in Ms-Excel 2016 and statistically analyzed using the statistical analysis software ms-Statistix 8.1. A two-way factorial ANOVA was performed to test the significance level whereas the means were separated using the least significance difference test at *p* < 0.05. The fertilizer application (FA) and the planting patterns (PP) were considered factors. Graphical and statistical software (GraphPad Prism 6.1) was used to determine the relationship of Pn with the grain yield, biomass dry matter, chlorophyll content, N content, PNUE, soluble sugars (i.e., sucrose, glucose, and fructose), and starch content of the maize crops under monocropping and intercropping systems.

## Results

### Physio-agronomic indices

The physio-agronomic indices of maize crops showed significant changes under different planting patterns and fertilization (Table 2). When compared to monocropping, intercropping significantly improved the physio-agronomic indices, such as plant height, stem diameter, grain yield, and biomass dry matter. However, these indices were more prominent under nitrogen fertilizer combined with foliar application of iron. Compared to monocropping, intercropping, the plant height (cm) increased by 3% at zero fertilizer application, 4% under nitrogen fertilizer application, and by 6% under nitrogen fertilization combined with foliar application of iron. Similarly, intercropping increased the stem diameter (mm) of maize by the respective values of 11, 13, and 15%. Moreover, intercropping increased the grain yield and biomass dry matter of maize by 24 and 8% without fertilizers application, 27 and 10% with nitrogen fertilization, and by 46 and 20% with nitrogen fertilization combined with foliar application of iron as compared to monocropping. Additionally, the LER value of the intercropping system was always greater than 1 in all treatment pots, indicating a yield advantage of intercropping over monocropping.

**TABLE 2:** Physio-agronomic indices of maize crops as influenced by nitrogen and iron fertilization under different planting patterns.

Fertilizer application (FA)	Planting pattern (PP)	Plant height (cm)	Stem diameter (mm)	Grain yield (g pot^–1^)	Biomass dry matter (g pot^–1^)	Land Equivalent Ratio (LER)
N0-Fe0	MM	245.75 ± 2.75 e	34.32 ± 2.64 c	102.75 ± 5.79 d	223.85 ± 11.05 e	
	IM	253.50 ± 3.41 d	38.20 ± 1.25 b	127.18 ± 7.51 b	242.15 ± 4.19 bc	1.23
N1-Fe0	MM	253.80 ± 2.64 d	37.12 ± 1.92 b	103.90 ± 7.15 cd	228.60 ± 9.01 de	
	IM	264.32 ± 3.17 b	41.95 ± 1.30 a	131.52 ± 2.53 b	250.73 ± 3.63 b	1.26
N1-Fe1	MM	258.70 ± 1.72 c	38.10 ± 1.32 b	112.30 ± 5.06 c	236.95 ± 4.51 cd	
	IM	274.15 ± 3.84 a	43.70 ± 1.92 a	163.69 ± 3.29 a	284.67 ± 7.31 a	1.45
**Significance levels**						
FA		0.00***	0.00***	0.00***	0.00***	
PP		0.00***	0.00***	0.00***	0.00***	
FA × PP		0.06^ns^	0.63^ns^	0.00***	0.00***	

The means with ± standard deviations (SD) having different lower-case letters are significantly different from each at the LSD test p ≤ 0.05 level of probability. FA, fertilizers application; PP, planting patterns; N0-Fe0, no nitrogen and iron application; N1-Fe0, nitrogen fertilizer without iron application; N1-Fe1, nitrogen fertilizer with the iron application. ***p ≤ 0.001, ^ns^*p* > 0.05.

### Leaf characteristics of maize crops

Different planting patterns and fertilizer applications significantly (*p* < 0.05) influenced the different leaf traits of the maize crops, but had minimal effects on the specific leaf mass (LMA) (Table 3). It was noticed that intercropping significantly improved the different leaf traits (i.e., number of leaves per plant, leaf area, leaf dry mass, leaf N content, and N content per unit area of maize crops) when compared to monocropping. However, these traits were more pronounced under different fertilization. Compared to monocropping, intercropping increased the number of leaves per plant by 13% without fertilization, 11% with nitrogen fertilization, and 20% with nitrogen fertilization combined with foliar application of iron. Likewise, under intercropping, there were respective increases by the values of 3, 3, and 6% for maize leaf area and 5, 7, and 9% for leaf dry mass. Moreover, intercropping increased the leaf N content and N content per unit area (N_area_) of maize crops by 7 and 13% without fertilization, 6 and 19% with nitrogen fertilization, and 8 and 20% with nitrogen fertilization combined with foliar application of iron, respectively.

**TABLE 3:** Leaf characteristics of maize crops as affected by nitrogen and iron fertilization under different planting patterns.

Fertilizers application (FA)	Planting patterns (PP)	No of leaves	Leaf area (cm^2^)	Leaf dry mass (g leaf^–1^)	Specific Leaf Mass (LMA) (g plant^–1^)	leaf N content (%)	N_Mass_ (g kg^–1^)	N_area_ (mg cm^2^)
N0-Fe0	MM	9.70 ± 0.82 c	279.12 ± 6.72 d	1.99 ± 0.08 c	0.71	2.81 ± 0.08 d	5.60 ± 0.19 e	4.01 ± 0.36 e
	IM	11.00 ± 0.70 bc	288.35 ± 6.47 c	2.08 ± 0.09 b	0.72	3.00 ± 0.12 bc	6.26 ± 0.33 cd	4.53 ± 0.33 cd
N1-Fe0	MM	11.00 ± 1.01 bc	288.81 ± 3.44 c	2.03 ± 0.10 c	0.70	2.92 ± 0.11 cd	5.95 ± 0.26 de	4.20 ± 0.39 de
	IM	12.25 ± 0.82 ab	296.81 ± 3.29 b	2.18 ± 0.05 b	0.73	3.10 ± 0.12 ab	6.79 ± 0.24 b	5.00 ± 0.23 b
N1-Fe1	MM	11.25 ± 0.82 b	291.40 ± 1.51 bc	2.15 ± 0.05 b	0.74	3.01 ± 0.11 bc	6.50 ± 0.28 bc	4.81 ± 0.29 bc
	IM	13.50 ± 0.51 a	310.13 ± 5.26 a	2.32 ± 0.04 a	0.75	3.25 ± 0.06 a	7.62 ± 0.14 a	5.76 ± 0.12 a
**Significance**								
FA		0.00***	0.00***	0.00***	0.13^ns^	0.00***	0.13^ns^	0.00***
PP		0.00***	0.00***	0.00***	0.14^ns^	0.00***	0.14^ns^	0.00***
FA × PP		0.47^ns^	0.08^ns^	0.46 *^ns^*	0.73^ns^	0.83^ns^	0.21^ns^	0.38^ns^

The means in the table with ± standard deviations (SD) having different lower-case letters are significantly different from each at the LSD test p ≤ 0.05 level of probability. FA, fertilizers application; PP, planting patterns; N0-Fe0, no nitrogen and iron application; N1-Fe0, nitrogen fertilizer without iron application; N1-Fe1, nitrogen fertilizer with the iron application. ***p ≤ 0.001, ^ns^p > 0.05.

### Chlorophyll, photosynthetic activities, and photosynthetic nitrogen use efficiency

**FIGURE 3:**
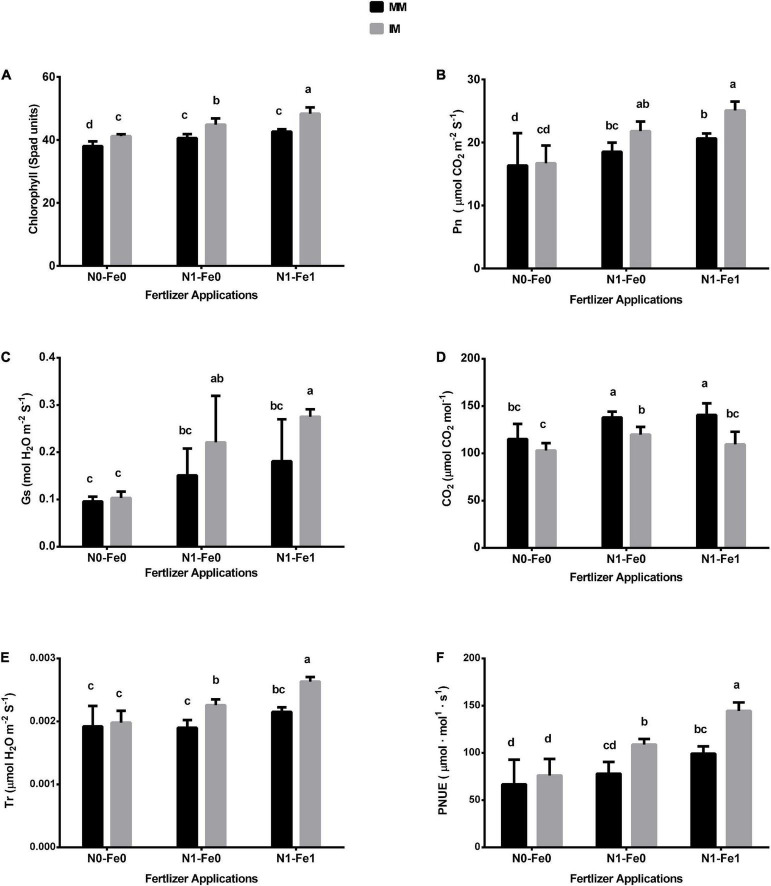
Chlorophyll **(A)**, Pn **(B)**, Gs **(C)**, CO_2_ **(D)**, Tr **(E)**, and PNUE **(F)** of the maize crops as influenced by different fertilizers and planting patterns. The column graphs with SD bars having dissimilar lower case letters are significantly different from each other at the LSD test (*p* < 0.05). MM, maize mono-cropping; IM, maize intercropping; Pn, photosynthetic rate; Gs, stomatal conductance; CO_2_, intercellular carbon dioxide; Tr, transpiration rate; PNUE, photosynthetic nitrogen use efficiency; N0-Fe0, no nitrogen and iron fertilization; N1-Fe0, nitrogen fertilization without iron foliation; N1-Fe1, nitrogen fertilization with iron foliation.

The chlorophyll and photosynthetic indices (i.e., Pn, Gs, Tr, and Ci) of maize varied significantly (*p* < 0.05) under different planting patterns and fertilizer applications (Figure 3). When compared with monocropping, intercropping increased the chlorophyll SPAD values and photosynthetic indices but these indices were further increased with the integration of different fertilizer treatments. For instance, intercropping increased the maize’s chlorophyll SPAD values by 8% without fertilization, 11% with nitrogen fertilization, and 13% with nitrogen fertilization coupled with foliar application of iron (Figure 3A). Furthermore, intercropping increased the Pn of maize crops by 4% without fertilization, 18% with nitrogen fertilization, and 21% with nitrogen fertilization combined with foliar application of iron (Figure 3B). Similarly, under intercropping, there were respective increases of 8, 46, and 52% for stomatal conductance (Gs) (Figure 3C), and 3, 19, and 22% for transpiration rate (Tr) (Figure 3D). Nonetheless, intercropping reduced the intercellular CO_2_ (Ci) by 11% without fertilization, 13% with nitrogen fertilization, and 22% with nitrogen fertilization combined with the iron foliar application (Figure 3E). In contrast, under intercropping, there was increased PNUE by 14, 39, and 45% without fertilization, with nitrogen fertilization, and with nitrogen fertilization combined with the iron foliar application, respectively (Figure 3F).

### Enzymes activities of maize leaf

**FIGURE 4:**
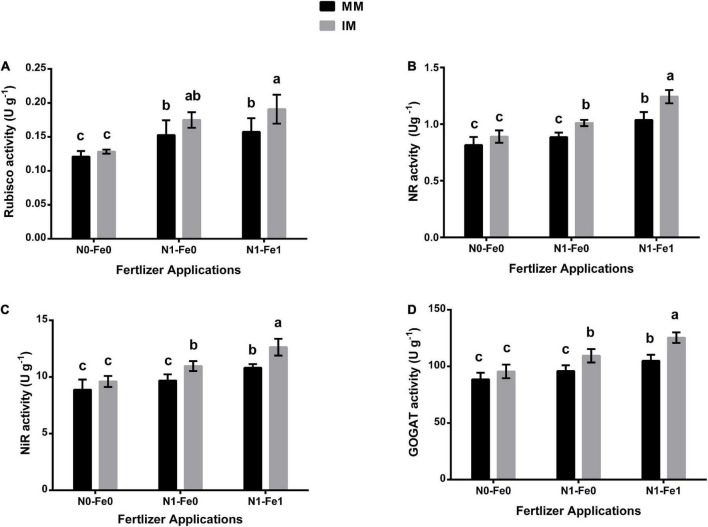
Enzymatic activities such as Rubisco activity **(A)**, NR activity **(B)**, NiR activity **(C)**, and GOGAT activity **(D)** of maize leaf as influenced by different fertilizer and planting patterns. The column graphs with SD bars having dissimilar lower case letters are significantly different from each other at the LSD test (*p* < 0.05). MM, maize mono-cropping; IM, maize intercropping; NR, nitrate reductase; NiR, nitrite reductase; GOGAT, glutamate synthase; N0-Fe0, no nitrogen and iron fertilization; N1-Fe0, nitrogen fertilization without iron foliation; N1-Fe1, nitrogen fertilization with iron foliation.

The photosynthetic and nitrogen metabolism-associated enzymes for maize indicated significant changes under different planting patterns and fertilization (Figure 4). When compared with monocropping, intercropping significantly increased these enzyme activities of the maize crops. However, these enzymes were enhanced further when intercropping was practiced with different fertilizer applications. It was noticed that intercropping increased the Rubisco activity of maize crops by 6% without fertilization, 14% with nitrogen fertilization, and 21% with nitrogen fertilization combined with foliar application of iron (Figure 4A). Similarly, intercropping increased the NR activity by 9% without fertilization, 14% with nitrogen fertilization, and 20% with nitrogen fertilization combined with foliar application of iron (Figure 4B). Moreover, under intercropping, there was increased NiR activity by 8, 13, and 17% without fertilization, with nitrogen fertilization, and with nitrogen fertilization combined with the iron foliar application, respectively (Figure 4C). A similar observation was made for GOGAT activity with the index increasing by 8% without fertilization, 14% with nitrogen fertilization, and 19% with nitrogen fertilization combined with foliar application of iron (Figure 4D).

### Sugar and starch content of maize crops

**FIGURE 5:**
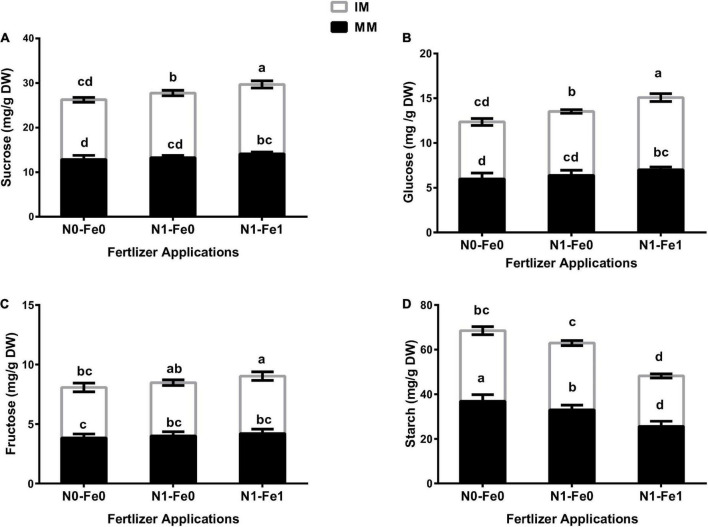
Soluble sugars such as sucrose **(A)**, glucose **(B)**, fructose **(C)**, and starch **(D)** content of the maize crops as influenced by different fertilizer and planting patterns. The column graphs with SD bars having lower case letters are significantly different from each other at the LSD test (*p* < 0.05). MM, maize mono-cropping; IM, maize intercropping; N0-Fe0, no nitrogen and iron fertilization; N1-Fe0, nitrogen fertilization without iron foliation; N1-Fe1, nitrogen fertilization with iron foliation.

The sugar content (i.e., sucrose, glucose, and fructose) and starch content of maize varied significantly (*p* < 0.05) under different planting patterns and fertilizer applications (Figure 5). However, the changes in sugars and starch contents were more evident under different fertilization. The results showed that although intercropping enhanced maize’s sugar content, it reduced the starch content. There was higher sucrose content of 4% without fertilization, 9% with nitrogen fertilization, and 11% with nitrogen fertilization combined with foliar application iron under intercropping compared with monocropping (Figure 5A). Moreover, intercropping increased the glucose content by 7% without fertilization, 12% with nitrogen fertilization, and 15% with nitrogen fertilization combined with foliar application of iron (Figure 5B). Besides, intercropping increased the fructose content by 11% without fertilization, 12% with nitrogen fertilization, and 15% with nitrogen fertilization combined with foliar application of iron (Figure 5C). However, intercropping reduced the starch content by 14% without fertilization, 9% with nitrogen fertilization, and 11% with nitrogen fertilization coupled with foliar iron application (Figure 5D).

### Regression analysis

**FIGURE 6:**
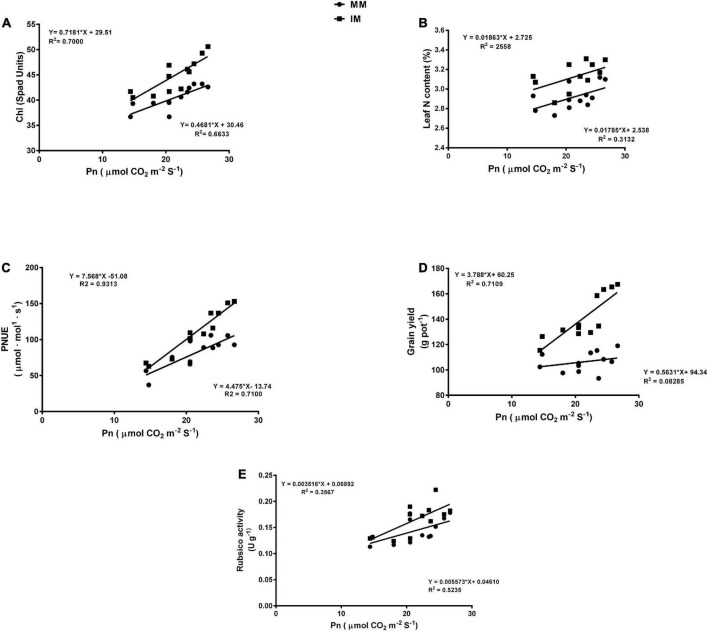
Regression analysis of the photosynthetic rate with chlorophyll (Chl) **(A)**, leaf N content **(B)**, PNUE **(C)**, grain yield **(D)**, and biomass yield **(E)** of the maize crops of maize during mono-cropping and intercropping. MM, maize mono-cropping; IM, maize intercropping.

**FIGURE 7:**
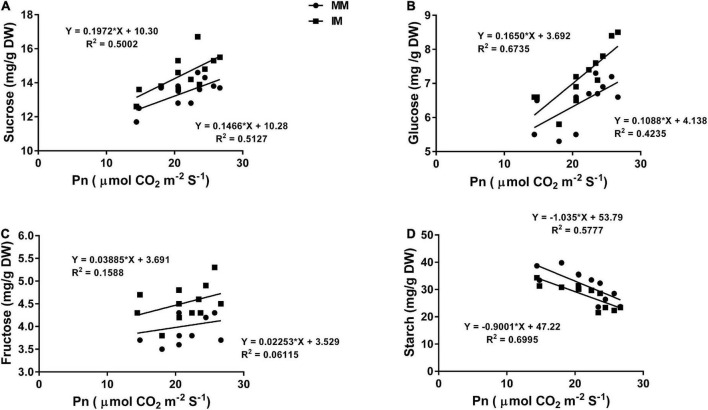
Regression analysis of the photosynthetic rate with sucrose **(A)**, glucose **(B)**, fructose **(C)**, and starch **(D)** of maize leaf during mono-cropping and intercropping. MM, maize mono-cropping; IM, maize intercropping.

**FIGURE 8:**
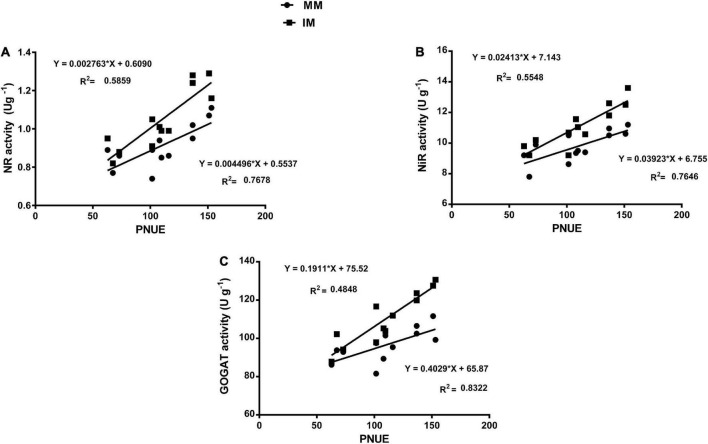
Regression analysis of the photosynthetic nitrogen use efficiency with NR activity **(A)**, NiR activity **(B)**, and GOGAT activity **(C)** of maize leaf during mono-cropping and intercropping. MM, maize mono-cropping; IM, maize intercropping; PNUE, photosynthetic nitrogen use efficiency; NR, nitrate reductase; NiR, nitrite reductase; GOGAT, glutamate synthase.

The linear regression analysis showed a significantly strong relationship (∼*r*>) of the Pn with the chlorophyll content (Figure 6A), leaf N content (Figure 6B), PNUE (Figure 6C) grain yield (Figure 6D), rubisco activity (Figure 6E), sucrose content (Figure 7A), glucose content (Figure 7B), and fructose content of maize crops (Figure 7C). Similarly, regression of PNUE with nitrogen metabolic enzymes showed significant strong relationships (Figure 8). However, the starch content was negatively correlated with the Pn (Figure 7D). Further, the results indicated that PNUE was significantly and positively correlated with nitrogen metabolic enzymes, such as NR (Figure 8A), NiR (Figure 8B), and GOGAT (Figure 8C).

## Discussion

In general, intercropping is practiced to improve crops yield and better utilize the available natural resources (i.e., water, nutrients, light, and land) (Kheroar and Patra, 2013; Latati et al., 2013; Nasar, 2018; Yang et al., 2018; Maitra et al., 2020). However, due to the differences in plant height in cereal–legume intercropping systems, the companion crops compete for solar radiation and capture sunlight in different directions. Such a phenomenon certainly changes the chlorophyll SPAD values and photosynthesis of the companion plants, with a direct effect on growth and yield (Nasar et al., 2020a). Moreover, the underlying interspecific competition for nutrients makes the use of the post-intercropping nitrogen more complicated, which adversely affects the plant nutrients uptake from the soil and its transport within the plant, thereby reducing the nutrient content in plant leaves. As a result, the plant cannot use the available nutrients more efficiently (Nasar et al., 2021; Nyawade et al., 2021). However, these negative effects of the intercropping system can be effectively reduced with nitrogen and iron fertilization. Nitrogen is the key component of chlorophyll content, enzyme content, and enzymatic activity of plant leaves (Evans, 1983; Pan et al., 2021). Iron, moreover, plays an important role in plant chlorophyll and photosynthesis, which gives plants oxygen and healthy green coloration (Wang et al., 2017). This is why iron-deficient plant shows chlorosis or a silky yellow color on their leaves; thus, iron is a crucial element for plant growth and development. In this study, we found that intercropping significantly increased the physio-agronomic indices of maize when compared to monocropping. However, these indices were further improved when intercropping was treated with nitrogen fertilizer combined with iron foliar applications. The improved physio-agronomic indices in intercropping were mainly because of the better utilization of the available natural resources (i.e., land, water, light, and nutrients) (Shao et al., 2020; Soratto et al., 2022). The previous results of maize–alfalfa intercropping supported our findings (Borghi et al., 2012; Nasar et al., 2020a; Shao et al., 2020). In another study iron foliar application either alone or in combination with nitrogen has also been reported to significantly improved the growth and yield of maize crops during intercropping with soybean (Dragicevic et al., 2015; Reddy et al., 2020).

Changes in the chlorophyll and photosynthetic characteristics can induce changes in the physiology and productivity of the plant (Mandal and Dutta, 2020; Shah et al., 2021a). It is believed that the changes in the chlorophyll content are expected to bring changes in the photosynthetic activities of the plant (Ahmad et al., 2013; Seleiman et al., 2021). Moreover, the changes in plant leaf enzyme activities (i.e., rubisco activity) could also bring changes in the photosynthetic activities of the plant (Wang et al., 2017; Shah et al., 2017). This study demonstrated that intercropping induced changes in the chlorophyll of the maize crops and rubisco activity, thereby enhancing its photosynthetic characteristics. However, these changes in the chlorophyll, rubisco activity, and photosynthetic activities of maize were more evident when intercropping was practiced under nitrogen fertilization combined with foliar application of iron. There could be several reasons for these changes, (i) the complementary interactions of intercrops, where one plant promotes the growth, survival, and fitness of its counterpart plant (Zhang et al., 2014; Nasar et al., 2020a), (ii) nitrogen fertilization, which is the key component of chlorophyll content, enzyme content, and enzymatic activity of the plant leaves (Evans, 1983; Pan et al., 2021), and (iii) Fe foliation or its improved nutrition of the plant caused by the underlying mechanisms in the intercropping system helps improve the chlorophyll content and rubisco activity, thereby promoting the photosynthesis of the plant (Geider and La Roche, 1994; Zuo et al., 2003; Jiang et al., 2007). Similar results were also reported in maize–alfalfa intercropping (Ning et al., 2018; Nasar et al., 2020a) and maize–peanut intercropping (Jiao et al., 2013).

It is also well known that different leaf characteristics of the plant are the main contributing factors affecting the chlorophyll and photosynthetic characteristics (Zhang et al., 2016; Zhu et al., 2018). Among the traits, the specific leaf area plays a significant role in the improvement of chlorophyll and photosynthesis (Zhang Y. et al., 2017). However, the leaf N content, leaf N_mass_, and N_area_ contribute to the improvement of plant leaf N content, thereby enhancing its PNUE (Da-yong et al., 2012). In this study, we found that intercropping significantly improved the different leaf characteristics, such as the number of leaves, leaf area, leaf dry mass, leaf N content, and N_area_ of the maize crops, as compared to monocropping. However, these indices were more pronounced under nitrogen fertilization combined with foliar application of iron. This might be attributed to the soybean facilitating the translocation of the fixed N and other nutrients to their corresponding maize crops during intercropping (Shao et al., 2020). In addition, such a phenomenon could be ascribed to the productive utilization of the available natural resources (i.e., water, light, nutrients, and land) (Latati et al., 2016; Gitari et al., 2020; Nasar et al., 2020b; Raza et al., 2021). Also, nitrogen and iron are the two important elements known for their major role in plant growth and development; thus, their application could further improve the leaf characteristics of maize (Nasar and Shah, 2017; Hu et al., 2018). Moreover, the improved leaf N content, leaf N_mass_, and N_area_ resulted in an improved PNUE of maize crops during intercropping (Zhang et al., 2014; Nasar et al., 2021). Consistent with our findings, several other studies have shown similar results in different cereal–legumes intercropping (Zhang et al., 2014; Raza et al., 2019b; Nasar et al., 2020a,^2021^). Furthermore, the enzyme’s cofactor of plant leaves, such as rubisco enzyme, NR, NiR, and GOGAT, are interrelated plant enzymes, which potentially contributes to the plant photosynthetic activities and nitrogen assimilation (i.e., uptake, translocation, and utilization), thereby enhancing the plant leaf nutrient utilization efficiency (PNUE) (Wang et al., 2012, 2017).

In this study, we found that intercropping increased the rubisco activity and the nitrogen assimilatory enzyme activity, such as NR, NiR, and GOGAT of the maize crops. The improved rubisco activity in the intercropping system could be due to the improved light conditions or strong light adaptability of the intercropping system (Boussadia et al., 2010). However, the changes in the nitrogen assimilatory enzymes might be due to the underlying nutrient facilitation by the legume crops to its corresponding cereal crops (Zhang H. et al., 2017) or could be due to the root releasing chemicals or root exudation (Thilakarathna et al., 2016). Nevertheless, these enzymatic activities were more evident when intercropping was practiced with nitrogen fertilization combined with foliar application of iron. The increased nitrogen assimilatory enzymes such as NR, NiR, and GOGAT activity in the intercropping system resulted in an improved PNUE of the maize crops (Vagusevičienė et al., 2013). These results are also consistent with the findings of Jiao et al. (2013) who stated that maize–peanut intercropping showed improved efficiency of photosynthetic activities, PNUE, and yield in maize crops under adequate nitrogen fertilization. In another maize–peanut intercropping study, it was shown that intercropping under appropriate nitrogen fertilization improved the Pn and PNUE, thereby enhancing the maize yield (Zhu et al., 2018).

Plant soluble sugar and starch content are important indicators describing the physiological behavior of plants (Oliveira et al., 2022). Any changes in such components in a plant can bring changes in the physiological and morphological indices (Qi et al., 2021). In this study, we found that intercropping significantly improved the plant’s soluble sugar content, but reduced its starch content. However, the improvement in sugar content was more evident when intercropping was practiced with nitrogen fertilization combined with foliar application of iron. The increased soluble sugar content in maize leaves under intercropping was mainly due to the changes in the photosynthetic efficiencies because sugar is the resultant product of photosynthesis (Yoon et al., 2019). In the same way, nitrogen and iron are the two key elements known for their involvement in the improvement of chlorophyll and photosynthesis efficiencies of the plant, which results in increased sugar content in plant leaves (Ning et al., 2018; Karimi et al., 2019). However, starch as a storage carbohydrate an important indicator for plant growth does not show any regulatory activities and can change in different growth stages (Ning et al., 2018). The decrease in starch content might be due to the starch synthesis competition with sucrose synthesis for Triose-Pi, which is a shared substrate for the two biochemical reactions (Stitt and Zeeman, 2012). Similar findings were observed in a maize–soybean intercropping system, where intercropping increased the soluble sugars compared to monocropping (Liu et al., 2017). It has also been shown that the combined application of nitrogen and iron can significantly increase the soluble sugar content in plant leaves but reduce the starch content (Karimi et al., 2019), which confirmed our results.

Taken together, our findings suggest that maize–soybean intercropping under optimal fertilization can improve the chlorophyll, photosynthetic activities, and associated enzymes, thereby enhancing the growth, yield, and PNUE of the maize crops. However, future research is still needed to explore more of the facts about the photosynthetic nitrogen use efficiency, particularly under the intercropping system. It will be more interesting to see the effect of nitrogen fertilization coupled with foliar application of iron and molybdenum on the photosynthetic activities and PNUE of the plants under intercropping given that both iron and molybdenum are well known for their role in photosynthetic and nitrogen metabolisms pathways. Moreover, it could also be much better to explore the key genes related to the photosynthetic activities and nitrogen assimilation pathways within the intercropping system.

## Conclusion

The current findings indicated that intercropping significantly enhanced the physio-agronomic indices as compared to monocropping. However, these indices were pronounced when intercropping was practiced with nitrogen fertilization combined with foliar application of iron. Moreover, intercropping under the same fertilization regimes improves the chlorophyll content, photosynthetic activities, its related leaf traits, and enzymatic activities. Furthermore, intercropping increased the enzymatic activities of nitrogen metabolism in maize crops, particularly under nitrogen fertilization combined with foliar application of iron. Such improvement of photosynthetic activities and enzymatic activities of maize crops coupled with fertilizer application resulted in improved photosynthetic nitrogen use efficiency and soluble sugar content, which eventually lead to better growth and higher yield in intercropping than monocropping. Thus, this suggests that intercropping under optimal nitrogen fertilization coupled with the iron foliar application could be vital for improving the leaf chlorophyll, photosynthetic characteristics, its related enzymes, nitrogen use efficiency, and crops yield.

## Data availability statement

The raw data supporting the conclusions of this article will be made available by the authors, without undue reservation.

## Author contributions

JN: conceptualization, methodology, and writing—original draft. G-YW: data curation. SA: formal analysis. MZ: resources. IM: software. X-BZ: supervision. HG, MA, SF, MK, NA, GA and MH: writing—review and editing. All authors contributed to the article and approved the submitted version.

## References

[B1] Abadia J. Nishio J. N. Terry N. (1986). Chlorophyll-protein and polypeptide composition of Mn-deficient sugar beet thylakoids. *Photosynth. Res.* 7 237–245. 10.1007/BF00014677 24443120

[B2] Ahmad I. Cheng Z. Meng H. Liu T. Nan W. C. Khan M. A. (2013). Effect of intercropped garlic (*Allium sativum*) on chlorophyl contents, photosynthesis and antioxidant enzymes in pepper. *Pakistan J. Bot.* 45 1889–1896.

[B3] Al-Dalain S. A. (2009). Effect of intercropping of Zea Maize with potato *Solanum tuberosum*, L. on potato growth and on the productivity and land equivalent ratio of potato and Zea maize. *Agric. J.* 4 164–170.

[B4] Borghi É Crusciol C. A. C. Nascente A. S. Mateus G. P. Martins P. O. Costa C. (2012). Effects of row spacing and intercrop on maize grain yield and forage production of palisade grass. *Crop Pasture Sci.* 63 1106–1113. 10.1071/CP12344

[B5] Borlotti A. Vigani G. Zocchi G. (2012). Iron deficiency affects nitrogen metabolism in cucumber (*Cucumis sativus L*.) plants. *BMC Plant Biol.* 12:189. 10.1186/1471-2229-12-189 PMC3539955 23057967

[B6] Boussadia O. Steppe K. Zgallai H. Ben El Hadj S. Braham M. Lemeur R. (2010). Effects of nitrogen deficiency on leaf photosynthesis, carbohydrate status and biomass production in two olive cultivars “Meski” and “Koroneiki.”. *Sci. Hortic. (Amsterdam)* 123 336–342. 10.1016/j.scienta.2009.09.023

[B7] Da-yong L. Zhi-an Z. Dian-jun Z. Li-yan j. yuan-li w. (2012). comparison of net photosynthetic rate in leaves of soybean with different yield levels. *J. Northeast Agric. Univ. (English Ed.)* 19 14–19.

[B8] Derviş B. Mei P. P. Gui L. G. Wang P. Huang J. C. Long H. Y. (2018). Differences in maize physiological characteristics, nitrogen accumulation, and yield under different cropping patterns and nitrogen levels. *Field Crop. Res.* 45 33–40. 10.1016/j.fcr.2018.04.004

[B9] Dragicevic V. Oljaca S. Stojiljkovic M. Simic M. Dolijanovic Z. Kravic N. (2015). Effect of the maize-soybean intercropping system on the potential bioavailability of magnesium, iron and zinc. *Crop Pasture Sci.* 66 1118–1127. 10.1071/CP14211

[B10] Du Y. Zhao Q. Chen L. Yao X. Zhang W. Zhang B. (2020). Effect of drought stress on sugar metabolism in leaves and roots of soybean seedlings. *Plant Physiol. Biochem.* 146 1–12. 10.1016/j.plaphy.2019.11.003 31710920

[B11] Ehrmann J. Ritz K. (2013). Plant: Soil interactions in temperate multi-cropping production systems. *Plant Soil* 376 1–29. 10.1007/s11104-013-1921-8

[B12] Evans J. R. (1983). Nitrogen and photosynthesis in the flag leaf of wheat (*Triticum aestivum* L.). *Plant Physiol.* 72 297–302. 10.1104/pp.72.2.297 16662996 PMC1066227

[B13] Geider R. J. La Roche J. (1994). The role of iron in phytoplankton photosynthesis, and the potential for iron-limitation of primary productivity in the sea. *Photosynth. Res.* 39 275–301. 10.1007/BF00014588 24311126

[B14] Ghannoum O. Evans J. R. Wah S. C. Andrews T. J. Conroy J. P. Von Caemmerer S. (2005). Faster RuBisco is the key to superior nitrogen-use efficiency in NADP-malic enzyme relative to NAD-malic enzyme C_4_ grasses. *Plant Physiol.* 137 638–650. 10.1104/pp.104.054759 15665246 PMC1065364

[B15] Giersch C. Robinson S. P. (1987). Regulation of photosynthetic carbon metabolism during phosphate limitation of photosynthesis in isolated spinach chloroplasts. *Photosynth. Res.* 14 211–227. 10.1007/BF00032706 24430736

[B16] Gitari H. I Karanja N. N. Gachene C. K. K. Kamau S. Sharma K. Schulte-Geldermann E. (2018). Nitrogen and phosphorous uptake by potato (*Solanum tuberosum* L.) and their use efficiency under potato-legume intercropping systems. *Field Crops Res.* 222 78–84. 10.1016/j.fcr.2018.03.019

[B17] Gitari H. I Nyawade S. O. Kamau S. Karanja N. N. Gachene C. K. K. Raza M. A. (2020). Revisiting intercropping indices with respect to potato-legume intercropping systems. *Field Crops Res.* 258:107957. 10.1016/j.fcr.2020.107957

[B18] Hikosaka K. (2004). Interspecific difference in the photosynthesis-nitrogen relationship: Patterns, physiological causes, and ecological importance. *J. Plant Res.* 117 481–494. 10.1007/s10265-004-0174-2 15583974

[B19] Hu F. Tan Y. Yu A. Zhao C. Coulter J. A. Fan Z. (2018). Low N fertilizer application and intercropping increases N concentration in pea (*Pisum sativum* L.) grains. *Front. Plant Sci.* 9:1763. 10.3389/fpls.2018.01763 30555501 PMC6284027

[B20] Jiang C. D. Gao H. Y. Zou Q. Shi L. (2007). Effects of iron deficiency on photosynthesis and photosystem II function in soybean leaf. *J. Plant Physiol. Mol. Biol.* 33 53–60. 17287570

[B21] Jiao N. Y. Ning T. Y. Yang M. K. Fu G. Z. Yin F. Xu G. W. (2013). Effects of maize | | peanut intercropping on photosynthetic characters and yield forming of intercropped maize. *Acta Ecol. Sin.* 33 4324–4330. 10.5846/stxb201207311087

[B22] Karimi R. Koulivand M. Ollat N. (2019). Soluble sugars, phenolic acids and antioxidant capacity of grape berries as affected by iron and nitrogen. *Acta Physiol. Plant.* 41:117. 10.1007/s11738-019-2910-1

[B23] Kheroar S. Patra B. C. (2013). Advantages of maize-legume intercropping systems. *J. Agric. Sci. Techonol.* 3 733–744. 10.1371/journal.pone.0113984 25486249 PMC4259307

[B24] Kong D. X. Li Y. Q. Wang M. L. Bai M. Zou R. Tang H. (2016). Effects of light intensity on leaf photosynthetic characteristics, chloroplast structure, and alkaloid content of *Mahonia bodinieri* (Gagnep.) Laferr. *Acta Physiol. Plant.* 38:120. 10.1007/s11738-016-2147-1

[B25] Kuai J. Liu Z. Wang Y. Meng Y. Chen B. Zhao W. (2014). Waterlogging during flowering and boll forming stages affects sucrose metabolism in the leaves subtending the cotton boll and its relationship with boll weight. *Plant Sci.* 223 79–98. 10.1016/j.plantsci.2014.03.010 24767118

[B26] Latati M. Bargaz A. Belarbi B. Lazali M. Benlahrech S. Tellah S. (2016). The intercropping common bean with maize improves the rhizobial efficiency, resource use and grain yield under low phosphorus availability. *Eur. J. Agron.* 72 80–90. 10.1016/j.eja.2015.09.015

[B27] Latati M. Pansu M. Drevon J.-J. Ounane S. M. (2013). Advantage of intercropping maize (*Zea mays* L.) and common bean (*Phaseolus vulgaris* L.) on yield and nitrogen uptake in Northeast Algeria. *Int. J. Res. Appl. Sci.* 1 1–7.

[B28] Liu Q. L. Li J. F. Fan Y. F. Deng C. R. Yong T. W. Liu W. G. (2017). Dynamics of soluble sugar and nitrogen contents in the stem and grain of soybean under relay intercropping and monoculture conditions. *Acta Pratacult. Sin.* 26 113–119. 10.11686/cyxb2016174

[B29] Liu Z. Gao F. Yang J. Zhen X. Li Y. Zhao J. (2019). Photosynthetic characteristics and uptake and translocation of nitrogen in peanut in a wheat–peanut rotation system under different fertilizer management regimes. *Front. Plant Sci.* 10:86. 10.3389/fpls.2019.00086 30792727 PMC6374608

[B30] Liu Z. Gao J. Gao F. Liu P. Zhao B. Zhang J. (2018). Photosynthetic characteristics and chloroplast ultrastructure of summer maize response to different nitrogen supplies. *Front. Plant Sci.* 9:576. 10.3389/fpls.2018.00576 29765387 PMC5938403

[B31] Maitra S. Hossain A. Brestic M. Skalicky M. Ondrisik P. Gitari H. (2020). Intercropping system – A low input agricultural strategy for food and environmental security. *Agronomy* 11:343. 10.3390/agronomy11020343

[B32] Maitra S. Praharaj S. Hossain A. Patro T. S. S. K. Pramanick B. (2022). “Small millets: The next-generation smart crops in the modern era of climate change,” in *Omics of Climate Resilient Small Millets*, eds Pudake R. N. Solanke A. U. Sevanthi A. M. Rajendrakumar P. (Singapore: Springer), 10.1007/978-981-19-3907-5_1

[B33] Mandal R. Dutta G. (2020). From photosynthesis to biosensing: Chlorophyll proves to be a versatile molecule. *Sensors Int.* 1:100058. 10.1016/j.sintl.2020.100058

[B34] Nasar J. (2018). Intercropping promote sustainable agriculture and clean enviroment. *Biomed. J. Sci. Tech. Res.* 4:2018. 10.26717/bjstr.2018.04.0001018

[B35] Nasar J. Shah Z. (2017). Effect of iron and molybdenum on yield and nodulation of lentil. *J. Agric. Biol. Sci.* 12 332–339.

[B36] Nasar J. Khan W. Khan M. Z. Gitari H. I. Gbolayori J. F. Moussa A. A. (2021). Photosynthetic activities and photosynthetic nitrogen use efficiency of maize crop under different planting patterns and nitrogen fertilization. *J. Soil Sci. Plant Nutr.* 21 2274–2284. 10.1007/s42729-021-00520-1

[B37] Nasar J. Shao Z. Gao Q. Zhou X. Fahad S. Liu S. (2020b). Maize-alfalfa intercropping induced changes in plant and soil nutrient status under nitrogen application. *Arch. Agron. Soil Sci.* 68 151–165. 10.1080/03650340.2020.1827234

[B38] Nasar J. Shao Z. Arshad A. Jones F. G. Liu S. Li C. (2020a). The effect of maize–alfalfa intercropping on the physiological characteristics, nitrogen uptake and yield of maize. *Plant Biol.* 22 1140–1149. 10.1111/plb.13157 32609937

[B39] Ning P. Yang L. Li C. Fritschi F. B. (2018). Post-silking carbon partitioning under nitrogen deficiency revealed sink limitation of grain yield in maize. *J. Exp. Bot.* 69 1707–1719. 10.1093/jxb/erx496 29361032 PMC5888971

[B40] Noor Shah A. Wu Y. Iqbal J. Tanveer M. Bashir S. Ur Rahman S. (2021). Nitrogen and plant density effects on growth, yield performance of two different cotton cultivars from different origin. *J. King Saud Univ. Sci.* 33 101512. 10.1016/j.jksus.2021.101512

[B41] Nyawade S. Gitari H. I. Karanja N. N. Gachene C. K. Schulte-Geldermann E. Sharma K. (2020). Enhancing climate resilience of rain-fed potato through legume intercropping and silicon application. *Front. Sustain. Food Sys.* 4:566345. 10.3389/fsufs.2020.566345

[B42] Nyawade S. Gitari H. I. Karanja N. N. Gachene C. K. Schulte-Geldermann E. Parker M. L. (2021). Yield and evapotranspiration characteristics of potato-legume intercropping simulated using a dual coefficient approach in a tropical highland. *Field Crops Res.* 274:108327. 10.1016/j.fcr.2021.108327

[B43] Ochieng’ I. O. Gitari H. I. Mochoge B. Rezaei-Chiyaneh E. Gweyi-Onyango J. P. (2021). Optimizing maize yield, nitrogen efficacy and grain protein content under different N forms and rates. *J. Soil Sci. Plant Nut.* 21 1867–1880. 10.1007/s42729-021-00486-0

[B44] Oliveira S. L. Crusciol C. A. C. Rodrigues V. A. Galeriani T. M. Portugal J. R. Bossolani J. W. (2022). Molybdenum foliar fertilization improves photosynthetic metabolism and grain yields of field-grown soybean and maize. *Front. Plant Sci.* 13:887682. 10.3389/fpls.2022.887682 35720532 PMC9199428

[B45] Pan S. Liu H. Mo Z. Patterson B. Duan M. Tian H. (2016). Effects of nitrogen and shading on root morphologies, nutrient accumulation, and photosynthetic parameters in different rice genotypes. *Sci. Rep.* 6:32148. 10.1038/srep32148 27557779 PMC4997252

[B46] Pan Y. Q. Tung S. A. Yang L. Wang Y. Zhou X. B. (2021). Effect of straw return and nitrogen application rate on the photosynthetic characteristics and yield of double-season maize. *J. Soil Sci. Plant Nutr.* 22 660–673. 10.1007/s42729-021-00676-w

[B47] Qi D. Li X. Pan C. Li J. Xu Y. Zhu J. (2021). Effect of nitrogen supply methods on the gas exchange, antioxidant enzymatic activities, and osmoregulation of maize (*Zea mays* L.) under alternate partial root-zone irrigation. *J. Soil Sci. Plant Nutr.* 21 2083–2095. 10.1007/s42729-021-00504-1

[B48] Raza M. A. Feng L. Y. Iqbal N. Ahmed M. Chen Y. K. Bin Khalid M. H. (2019a). Growth and development of soybean under changing light environments in relay intercropping system. *PeerJ* 7:e7262. 10.7717/peerj.7262 31372317 PMC6659667

[B49] Raza M. A. Feng L. Y. van der Werf W. Iqbal N. Khan I. Hassan M. J. (2019b). Optimum leaf defoliation: A new agronomic approach for increasing nutrient uptake and land equivalent ratio of maize soybean relay intercropping system. *Field Crop. Res.* 244:107647. 10.1016/j.fcr.2019.107647

[B50] Raza M. A. Feng L. Y. van der Werf W. Iqbal N. Khan I. Khan A. (2020). Optimum strip width increases dry matter, nutrient accumulation, and seed yield of intercrops under the relay intercropping system. *Food Energy Secur.* 9:e199. 10.1002/fes3.199

[B51] Raza M. A. Gul H. Wang J. Yasin H. S. Qin R. Khalid M. H. B. (2021). Land productivity and water use efficiency of maize-soybean strip intercropping systems in semi-arid areas: A case study in Punjab Province, Pakistan. *J. Cleaner Prod.* 308:127282. 10.1016/j.jclepro.2021.127282

[B52] Reddy D. Ganesh P. Saisravan A. Dawson J. (2020). Effect of nitrogen and iron levels on growth and yield of rabi hybrid maize (*Zea mays* L.). *Int. J. Curr. Microbiol. Appl. Sci.* 9 2297–2304. 10.20546/ijcmas.2020.911.275

[B53] Seleiman M. F. Aslam M. T. Alhammad B. A. Hassan M. U. Maqbool R. Chattha M. U. (2021). Salinity stress in wheat: Effects, mechanisms and management strategies. *Phyton Int. J. Exp. Bot.* 91 667–694. 10.32604/phyton.2022.017365

[B54] Shah A. N. Tanveer M. Abbas A. Yildirim M. Shah A. A. Ahmad M. I. (2021a). Combating dual challenges in maize under high planting density: Stem lodging and kernel abortion. *Front. Plant Sci.* 12:699085. 10.3389/fpls.2021.699085 34868101 PMC8636062

[B55] Shah A. N. Wu Y. Tanveer M. Hafeez A. Tung S. A. Ali S. (2021b). Interactive effect of nitrogen fertilizer and plant density on photosynthetic and agronomical traits of cotton at different growth stages. *Saudi J. Biol. Sci*. 28, 3578–3584. 10.1016/j.sjbs.2021.03.034 34121901 PMC8176129

[B56] Shah A. N. Yang G. Tanveer M. Iqbal J. (2017). Leaf gas exchange, source–sink relationship, and growth response of cotton to the interactive effects of nitrogen rate and planting density. *Acta Physiol. Plant*. 39:119. 10.1007/s11738-017-2402-0

[B57] Shao Z. Wang X. Gao Q. Zhang H. Yu H. Wang Y. (2020). Root contact between maize and alfalfa facilitates nitrogen transfer and uptake using techniques of foliar 15N-labeling. *Agronomy* 10:360. 10.3390/agronomy10030360

[B58] Soratto R. P. Perdoná M. J. Parecido R. J. Pinotti R. N. Gitari H. I. (2022). Turning biennial into biannual harvest: Long-term assessment of Arabica coffee–macadamia intercropping and irrigation synergism by biological and economic indices. *Food Energy Secur.* 11:e365. 10.1002/fes3.365

[B59] Stitt M. Zeeman S. C. (2012). Starch turnover: Pathways, regulation and role in growth. *Curr. Opin. Plant Biol.* 15 282–292. 10.1016/j.pbi.2012.03.016 22541711

[B60] Terry N. (1983). Limiting factors in photosynthesis: IV. Iron stress-mediated changes in light-harvesting and electron transport capacity and its effects on photosynthesis *in Vivo*. *Plant Physiol.* 71 855–860. 10.1104/pp.71.4.855 16662919 PMC1066134

[B61] Thilakarathna M. S. Mcelroy M. S. Chapagain T. Papadopoulos Y. A. Raizada M. N. (2016). Belowground nitrogen transfer from legumes to non-legumes under managed herbaceous cropping systems. A review. *Agron. Sustain. Dev.* 36:58. 10.1007/s13593-016-0396-4

[B62] Vagusevičienė I. Juchnevičienë A. Duchovskis P. (2013). The effect of nitrogen fertilizers on the changes of photosynthetic pigments in winter wheat. *Proc. Int. Sci. Conf. Rural Dev.* 6 258–261.

[B63] Wang Y. Y. Hsu P. K. Tsay Y. F. (2012). Uptake, allocation and signaling of nitrate. *Trends Plant Sci.* 17 458–467. 10.1016/j.tplants.2012.04.006 22658680

[B64] Wang Y. Xu C. Li K. Cai X. Wu M. Chen G. (2017). Fe deficiency induced changes in rice (*Oryza sativa* L.) thylakoids. *Environ. Sci. Pollut. Res.* 24 1380–1388. 10.1007/s11356-016-7900-x 27783241

[B65] Wu Y. Gong W. Yang W. (2017). Shade inhibits leaf size by controlling cell proliferation and enlargement in soybean. *Sci. Rep.* 7:9259. 10.1038/s41598-017-10026-5 28835715 PMC5569092

[B66] Yang C. Fan Z. Chai Q. (2018). Agronomic and economic benefits of pea/maize intercropping systems in relation to n fertilizer and maize density. *Agronomy* 8 2–14. 10.3390/agronomy8040052

[B67] Yong T. W. Yang W. Y. Xiang D. B. Chen X. R. (2012). Effects of different cropping modes on crop root growth, yield, and rhizosphere soil microbes’ number. *Chinese J. Appl. Ecol.* 23 125–132. 22489489

[B68] Yoon H. Kang Y. G. Chang Y. S. Kim J. H. (2019). Effects of zerovalent iron nanoparticles on photosynthesis and biochemical adaptation of soil-grown *Arabidopsis thaliana*. *Nanomaterials* 9:1543. 10.3390/nano9111543 31671607 PMC6915611

[B69] Zhang H. Zeng F. Zou Z. Zhang Z. Li Y. (2017). Nitrogen uptake and transfer in a soybean/maize intercropping system in the karst region of southwest China. *Ecol. Evol.* 7 8419–8426. 10.1002/ece3.3295 29075459 PMC5648690

[B70] Zhang K. K. Zhou S. M. Zhang M. Shi S. S. Yin J. (2016). Effects of reduced nitrogen application and supplemental irrigation on photosynthetic characteristics and grain yield in high-yield populations of winter wheat. *Chinese J. Appl. Ecol.* 27 863–872. 10.13287/j.1001-9332.201603.006 29726192

[B71] Zhang X. Huang G. Zhao Q. (2014). Differences in maize physiological characteristics, nitrogen accumulation, and yield under different cropping patterns and nitrogen levels. *Chil. J. Agric. Res.* 74 326–332. 10.4067/S0718-58392014000300011 27315006

[B72] Zhang X. Huang G. Bian X. Zhao Q. (2013). Effects of nitrogen fertilization and root interaction on the agronomic traits of intercropped maize, and the quantity of microorganisms and activity of enzymes in the rhizosphere. *Plant Soil* 368 407–417. 10.1007/s11104-012-1528-5

[B73] Zhang Y. Wang J. Gong S. Xu D. Sui J. (2017). Nitrogen fertigation effect on photosynthesis, grain yield and water use efficiency of winter wheat. *Agric. Water Manag.* 179 277–287. 10.1016/j.agwat.2016.08.007

[B74] Zhong C. Jian S. F. Huang J. Jin Q. Y. Cao X. C. (2019). Trade-off of within-leaf nitrogen allocation between photosynthetic nitrogen-use efficiency and water deficit stress acclimation in rice (*Oryza sativa* L.). *Plant Physiol. Biochem.* 135 41–50. 10.1016/j.plaphy.2018.11.021 30500517

[B75] Zhu Q. L. Xiang R. Tang L. Long G. Q. (2018). Effects of intercropping on photosynthetic rate and net photosynthetic nitrogen use efficiency of maize under nitrogen addition. *Chinese J. Plant Ecol.* 42 672–680. 10.17521/cjpe.2018.0033

[B76] Zuo Y. Li X. Cao Y. Zhang F. Christie P. (2003). Iron nutrition of peanut enhanced by mixed cropping with maize: Possible role of root morphology and rhizosphere microflora. *J. Plant Nutr.* 26 2093–2110. 10.1081/PLN-120024267

